# A General Grid-Less Design Method for Location and Pressure Sensors with High Precision

**DOI:** 10.3390/s20247286

**Published:** 2020-12-18

**Authors:** Xiaobo Zhu, Xiong Cheng, Weidong Zhang, Jiale Gao, Yijie Dai, Wenhua Gu

**Affiliations:** School of Electronic and Optical Engineering, Nanjing University of Science and Technology, Xiaolingwei Street 200#, Nanjing 210094, China; zhuxiaobo@njust.edu.cn (X.Z.); orangebear@njust.edu.cn (X.C.); zwd@njust.edu.cn (W.Z.); gaojiale@njust.edu.cn (J.G.); dalbert2020@njust.edu.cn (Y.D.)

**Keywords:** grid-less planar pressure sensor, BP neural network, high-precision

## Abstract

Bionic electronic skin can accurately sense and locate surface pressure, which is widely demanded in many fields. Traditional electronic skin design usually relies on grid-architecture sensor arrays, requiring complex grid and interconnection arrangements as well as high cost. Grid-less planar sensors can solve the problem by using electrodes only at the edges, but they usually require the use of mapping software such as electrical impedance tomography to achieve high precision. In this work, a design method of high-precision grid-less planar pressure sensors based on the back-propagation (BP) neural network is proposed. The measurement precision of this method is demonstrated to be over two orders of magnitude higher than that of a grid-structure sensor array with the same electrode distribution density. Moreover, this method can be used for irregularly-shaped and non-uniform sensors, which further reduces the manufacturing difficulty and increases the application flexibility.

## 1. Introduction

As one of the representative flexible sensor systems, bionic electronic skin has wide applications in medical rehabilitation [1,2,3], motion imaging [4,5,6], virtual reality [7], Internet of Things [8], and other fields. One important feature of electronic skin for pressure sensing is the accurate mapping of both the magnitude and location of the pressure. High-precision pressure mapping usually relies on grid-architecture planar sensor arrays. A large number of densely arranged sensor elements are needed to achieve high localization precision, and each sensor element responds to the pressure applied on it independently [9,10,11,12]. These sensing elements must meet strict requirements, including positioning accuracy, uniformity, sensitivity, measurement range, and so on [13,14,15]. Even more, the accuracy of the active elements strongly relies on the tedious and complex pre-calibration process. The above requirements usually result in complex designs and expensive manufacturing processes [16,17].

Some researchers have proposed the design of grid-less planar sensors, which use a sheet of uniform sensing materials with a number of electrodes at the edges only, avoiding the complex grid architecture design intervening the sensing elements and the conducting wires, and using electrical impedance tomography (EIT) to measure the electrical signal changes between specific electrodes to reconstruct the pressure distribution [18,19,20,21,22,23,24]. EIT is an algorithm that reconstructs electromagnetic signals into physical information to realize three-dimensional imaging [20]. It is widely used in medical imaging, damage detection, and other fields [21,22]. However, this method also has certain limitations. First, professional commercial software as well as hardware support with high computing power are required. Secondly, this algorithm needs to be manually corrected based on the contact resistance of the electrode, the uniformity of the planar sensor, and other device parameters, and the calculation setup such as the boundary condition setting and mesh dividing also requires human intervention to ensure good convergence. Finally, the resolution of pressure and localization based on EIT is also mainly limited by the number of electrodes.

In this work, the back-propagation (BP) neural network is used to train the experimental data so as to achieve high-precision pressure and location sensing in a grid-less planar pressure sensor. This method not only avoids the complicated grid architecture and circuit design but also achieves surprisingly high precision using a small number of electrodes. This method can be a high-precision, cost-effective, strongly adaptable solution for electronic skins and many other applications.

The BP neural network [25] is a mathematical model that uses big data training to accurately predict the output data of a system at a given input without knowing the mapping relationship. It has been widely used in the fields of function approximations and pattern recognition and classification tasks [26,27,28]. In addition, a large number of research results have shown that Application Specific Integrated Circuits (ASICs) can be designed to realize integration of micro-systems based on different neural network characteristics, which is important for potential applications of micro sensor systems. The neural network has been used for grid-architecture pressure sensor arrays to improve the accuracy of pressure perception and positioning [29,30], but to the best of our knowledge, no such work on grid-less planar pressure sensors has been reported yet.

## 2. Pressure Sensor Fabrication and Measurement

In this work, grid-less piezoresistive planar pressure sensors were designed for verification of the proposed method using conductive silicone rubber as the sensing material. Conductive silicone rubber is a commonly used piezoresistive material, which has the characteristics of good stability, large measurement range, and easy fabrication for large area applications, but it also has disadvantages such as low sensitivity and large hysteresis.

**Figure 1:**
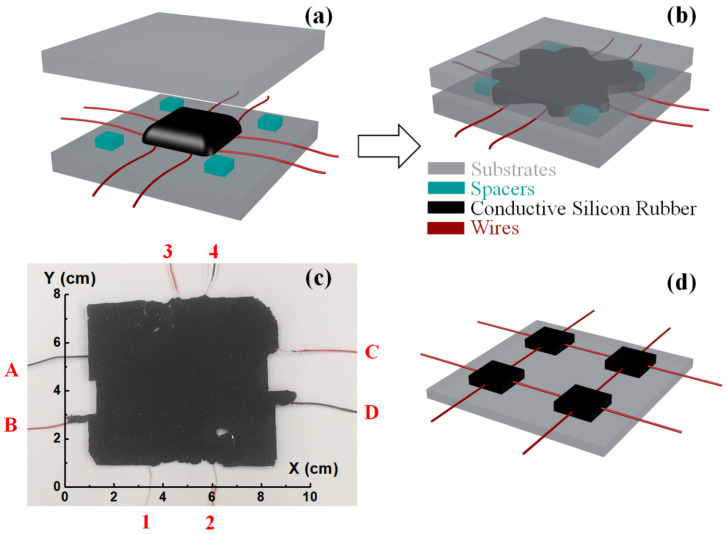
(**a**) Conductive rubber solution was dropped onto the center of the template, and 8 electrodes were embedded into the conductive rubber; (**b**) the conductive rubber was compressed into an irregularly-shaped planar structure; (**c**) picture of the fabricated sample with embedded and numbered electrodes; (**d**) the schematic diagram of a 2 × 2 traditional grid-architecture sensor array for comparison.

A home-made conductive silicon rubber sheet was used to fabricate the grid-less planar pressure sensor. All the processes were operated at room temperature. The first step was to prepare the conductive silicone rubber raw materials by adding together dimethyl silicone oil (0.5 g), ethyl orthosilicate (0.25 g), and dibutyltin dilaurate (0.125 g) into 5 g 107 silicone rubber and stirring well. Then, 1.5 g of carbon black powder (VXC-72, Cabot Corporation, Alpharetta, GA, USA) was added, and the conductive rubber solution was formed after stirring again. Then, the liquid conductive silicone rubber was compressed into a planar sheet and cured. Before compressing, metal wires with spiral shapes were inserted into the conductive silicon rubber as electrodes. Eight electrodes were used in this work, as shown in Figure 1a. In order to verify that a sensor with an irregular shape can also accurately sense the location and pressure, the conductive rubber was compressed using, not a fixed shape mold, but four 1 mm-thick glass spacers to constrain the thickness of the conductive rubber only. Thus, a planar pressure sensor with an irregular shape was fabricated, as shown in Figure 1b. Subsequently, the planar pressure sensor was cured at room temperature for about 24 h with 2000 kPa of pressure applied on it. Finally, a grid-less piezoresistive planar pressure sensor with embedded electrodes was obtained (Figure 1c). The area of the sensor was roughly 7.5 cm × 6.5 cm, and the distance between adjacent electrodes was about 2 cm. The schematic diagram of a 2 × 2 traditional grid-architecture sensor array is also shown in Figure 1d, which has a more complex structure with less sensing area but more interconnecting wires.

**Figure 2:**
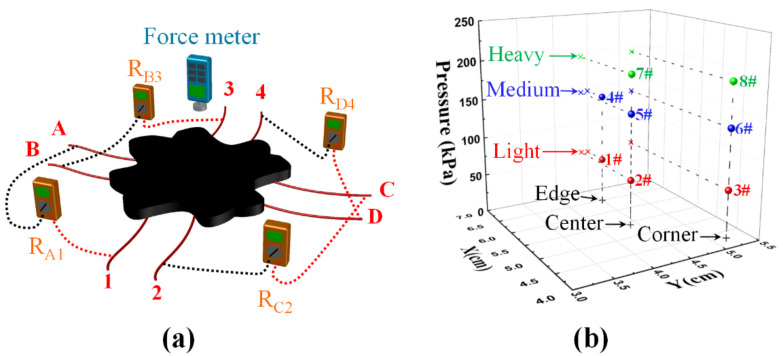
(**a**) Schematic diagram of the measurement system; (**b**) three-dimensional mapping of the pressure and localization of 8 typical data samples.

During the measurement, the electrodes were numbered and paired, as shown in Figure 2a, and the resistances between A1, B3, C2, and D4 were measured simultaneously with multimeters (Victor 88D) under different applied-pressure conditions, as the training inputs of the BP neural network. A force meter (SP-50 produced by Shanghai Siwei Instrument Manufacturing Co., Ltd, Shanghai, China) was used to apply different pressures at different locations of the sensor, and the pressures could be calculated from the force value shown on the force meter. The pressure along with the *X* and *Y* coordinates were recorded and used as the training outputs of the BP neural network, as shown in Figure 2a. In this work, 14 locations were randomly selected on the sensor surface, and 4–5 different pressures in the range of (56–260 kPa) were randomly applied at each location. A total number of 64 groups of input–output data were obtained as the training database to train the BP neural network model. In addition, another 8 groups of data were measured as test data samples to verify the accuracy of the model. The pressure and localization values of the 8 test data samples are recorded in Table 1, and the 3D mapping is shown in Figure 2b. For typicality, the 8 test data samples were distributed in 3 locations, corresponding to the edge (1#, 4#), center (2#, 5#, 7#), and corner (3#, 6#, 8#) of the sensor, respectively. The pressure values could be divided into 3 levels: light (1#, 2#, 3#), medium (4#, 5#, 6#) and heavy (7#, 8#). By analyzing the prediction performance of the neural network model in different areas and at different pressure levels, the pros and cons of the design method could be comprehensively analyzed.

## 3. The BP Neural Network

The BP neural network is composed of an input layer, a hidden layer, and an output layer, and each layer contains several neurons. The neurons in the input layer are only responsible for receiving the normalized input data and passing the data to the neurons in the next layer. The number of neurons *N_I_* is the dimension of the input vector (without considering the bias). The neurons in the hidden layer multiply or accumulate the data from the previous layer by weights and map the data to the input of the next layer using the activation function after adding the bias of the neuron. The number of neurons in the hidden layer *N_H_* is usually determined by experience [31]. The output layer is the last layer of the entire network and has the same function as the hidden layer, and the number of neurons in output layer *N_O_* is the same as the dimension of the output vector.

**Figure 3:**
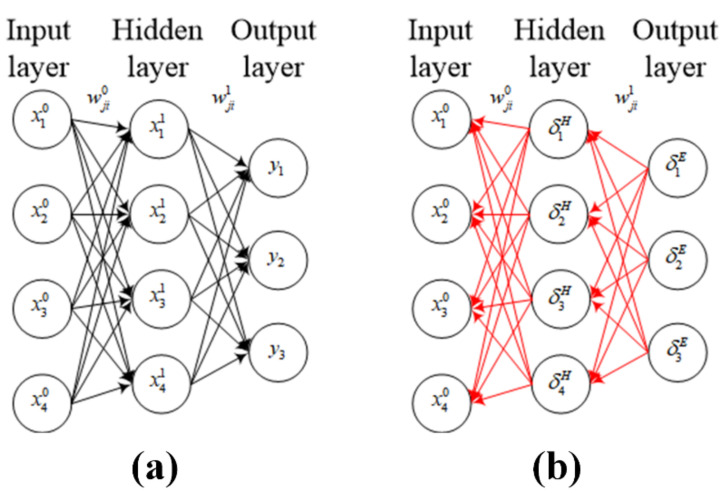
Schematic diagrams of (**a**) feed-forward and (**b**) feed-backward propagation processes of a 2-layer neural network.

Figure 3a shows the feed-forward process of a simple 2-layer network (the input layer is the *0*th layer). Here, $w_{ji}^{k}$ refers to the weight in the transferring from the *i*th neuron in the *k*th layer to the *j*th neuron in the *k + 1*th layer, and $b_{j}^{k}$ is the bias of the *j*th neuron in the *k + 1*th layer. We define $z_{j}^{k}$ and $x_{j}^{k}$ as the values of the *j*th neuron in the *k + 1*th layer before and after activation, respectively, and the feed-forward propagation process of the neural network can be expressed by the following formula:

(1) $$z_{j}^{l}=\sum_{i}w_{ji}^{0}x_{i}^{0}+b_{j}^{0}$$

(2) $$x_{j}^{1}=f(z_{j}^{1})$$

(3) $$z_{j}^{2}=\sum_{i}w_{ji}^{1}x_{i}^{1}+b_{j}^{1}$$

(4) $$y_{j}=f(z_{j}^{2})$$

where *y_j_* is the value of the *j*th vector in the output layer. The error between the network output and the real output is

(5) $$E=\frac{1}{2}\sum_{j=1}^{N_{O}}{\left(y_{j}-y_{j}^{\prime }\right)}^{2}.$$

Usually, the initial weights are randomly generated, and there is a large error between the output calculated by the network and the real value. Therefore, the error back propagation process shown in Figure 3b is required to adjust and update the weights so as to reduce the error until it finally converges to a low-enough level. Taking the network shown in Figure 3b as an example, we define $\delta_{j}^{E}$ and $\delta_{j}^{H}$ as the error of the *j*th neuron in the output layer and the hidden layer, respectively, and the error transfer formulae are as follows:

(6) $$\delta^{E}=(\nabla E)⊙f’(z^{2})$$

(7) $$\delta^{H}=({\left(w_{ji}^{1}\right)}^{T}\delta^{E})⊙f’(z^{1})$$

The corresponding updated formulae for weights and biases are

(8) $$w_{ji}^{1}=w_{ji}^{1}-\alpha \frac{\partial E}{\partial w_{ji}^{1}}=w_{ji}^{1}-\alpha x_{j}^{1}\delta_{i}^{E}$$

(9) $$w_{ji}^{0}=w_{ji}^{0}-\alpha \frac{\partial E}{\partial w_{ji}^{0}}=w_{ji}^{0}-\alpha x_{j}^{0}\delta_{i}^{H}$$

(10) $$b_{j}^{1}=b_{j}^{1}-\alpha \frac{\partial E}{\partial b_{j}^{1}}=\delta_{j}^{E}$$

(11) $$b_{j}^{0}=b_{j}^{0}-\alpha \frac{\partial E}{\partial b_{j}^{0}}=\delta_{j}^{H}.$$

Here the learning rate *α* is a hyperparameter, and the network can converge to different levels at different speeds by adjusting *α*. In this work, we used a typical 2-layer network, the number of neurons (*N_I_*, *N_H_*, *N_O_*) is (4, 4, 4), the computing platform is CPU (AMD Ryzen 7 4800H), the maximum number of epochs is 800 k, and the learning rate *α* is set to 10^−5^. It can be seen from the above setup that a very simple neural network and very limited resources are used, which can actually lead to very good results in this design, as shown below.

## 4. Results and Discussions

### 4.1. Experimental Results and Analysis

**Figure 4:**
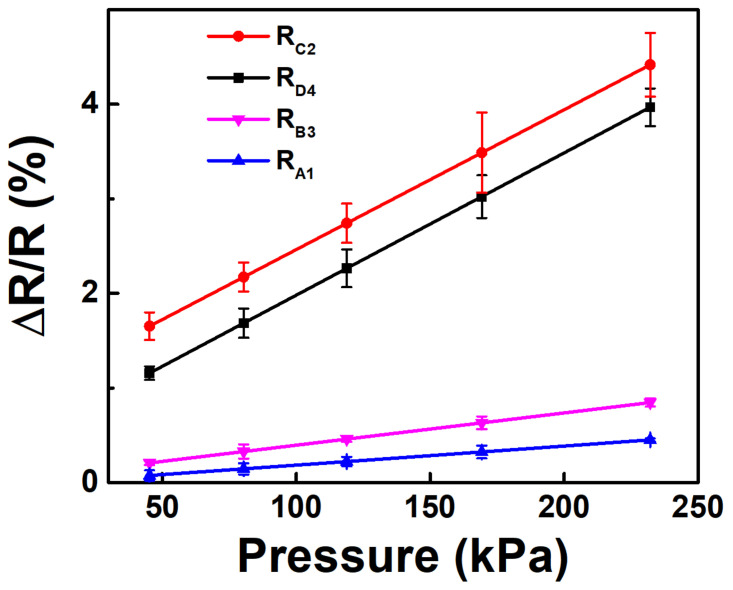
The relative resistance changes between the 4 pairs of electrodes at the location of (5.1 cm, 3.9 cm) under different pressures.

First, let us examine the piezo-resistance performance of the sensor itself without using the BP neural network. In the 64 groups of training data samples, we took different pressures applied on the location (*x* = 5.1 cm, *y* = 3.9 cm) as examples to show the piezoresistive characteristics between each pair of electrodes, as shown in Figure 4, in which the error bars indicate the differences between the measured data and the linear regression fitting data. The sensitivity *S* is defined as follows:

(12) $$S=\frac{\Delta R/R}{P}$$

where *P* is the pressure. The maximum sensitivity at this location is about 1.7 × 10^−5^ kPa^−1^. Compared to other piezoresistive pressure sensors, the sensitivity of this sample itself without using the BP neural network is relatively poor. The *R^2^* values of the linear regression fitting of the relative resistance change between the A1, B3, C2, and D4 electrodes are 0.975, 0.930, 0.962, and 0.984, respectively, which indicate relatively good linearity. In addition, the pressure range of the sample was found to be very large in the experiment, far exceeding the measurement range (300 kPa) of the force meter used in this work.

### 4.2. Analysis of the Neural Network Prediction Performance

The above-mentioned two-layer BP neural network reached a relatively high learning rate after 64 groups of data sample training, and could make good predictions of the pressure values and locations. The eight test data samples were used to check and evaluate the performance of the neural network prediction from different aspects, as shown below.

#### 4.2.1. Precision Analysis

**Figure 5:**
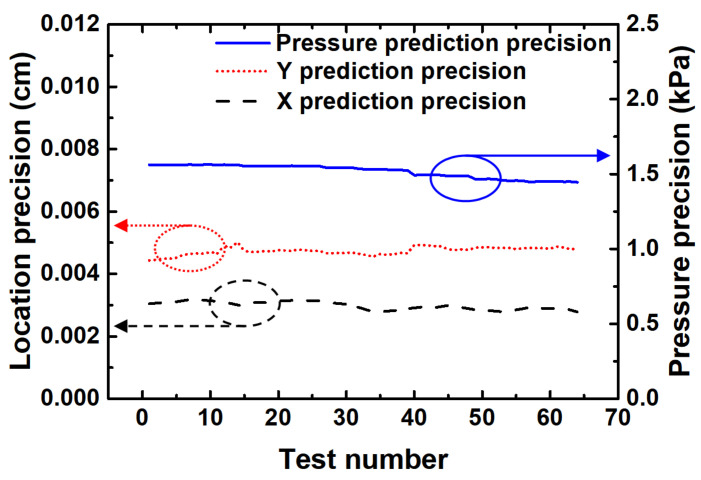
The precision of predicted *X*, *Y*, and *P*. The input precision is $dR_{A1}$ = $dR_{B3}$ = $dR_{C2}$ = $dR_{D4}$ = 0.1 Ω.

The prediction precision of the neural network model is calculated and illustrated in Figure 5. The three-dimensional space measurement precision is defined in the ISO230-6 standard as the square root of the total squared precisions in three directions (*x*, *y*, and *z*). In this work, the input signals of the neural network were the relative resistance change between four pairs of electrodes, which is a four-dimensional vector, and the output is a three-dimensional vector, including coordinates (*X* and *Y*) and pressure *P*. The input vector precision is $dR_{A1}$ = $dR_{B3}$ = $dR_{C2}$ = $dR_{D4}$ = 0.1 Ω, which is determined by the minimum measurement limit of the multimeter. The output precisions *dX*, *dY*, and *dP* for each training sample are calculated according to the following formula:

(13) $$\left\{\begin{array}{l} dX_{n}=\sqrt{X_{n}{\left(\frac{dR_{A1}}{R_{A1}}\right)}^{2}+X_{n}{\left(\frac{dR_{B3}}{R_{B3}}\right)}^{2}+X_{n}{\left(\frac{dR_{C2}}{R_{C2}}\right)}^{2}+X_{n}{\left(\frac{dR_{D4}}{R_{D4}}\right)}^{2}}\\ dY_{n}=\sqrt{Y_{n}{\left(\frac{dR_{A1}}{R_{A1}}\right)}^{2}+Y_{n}{\left(\frac{dR_{B3}}{R_{B3}}\right)}^{2}+Y_{n}{\left(\frac{dR_{C2}}{R_{C2}}\right)}^{2}+Y_{n}{\left(\frac{dR_{D4}}{R_{D4}}\right)}^{2}}\\ dP_{n}=\sqrt{P_{n}{\left(\frac{dR_{A1}}{R_{A1}}\right)}^{2}+P_{n}{\left(\frac{dR_{B3}}{R_{B3}}\right)}^{2}+P_{n}{\left(\frac{dR_{C2}}{R_{C2}}\right)}^{2}+P_{n}{\left(\frac{dR_{D4}}{R_{D4}}\right)}^{2}}\\ n=1,2,\cdots ,64 \end{array}\right.$$

It can be seen from Figure 5 that the prediction precisions of the neural network model at different locations and under different pressures were relatively stable, indicating that the model has good uniformity and linearity. The slight difference between the precisions in *X* coordinate and *Y* coordinate is mainly due to the fact that the sampling density in the y direction was higher than that in the *x* direction. The slight difference between the precisions in *X* coordinate and *Y* coordinate is probably due to the fact that the sampling ranges in the two directions were different. Our test equipment had a larger measurement range in the *x* direction than that in the *y* direction; thus, the sampling range in the *x* direction was larger than that in the *y* direction, and the prediction precision in the *X* coordinate was smaller than that in the *Y* coordinate consequently.

In general, the neural network prediction precision of the localization is smaller than 0.006 cm. For a traditional grid-structure planar sensor array with the same area (7.5 cm × 6.5 cm) and the same number of electrodes (2 × 2), the electrode distribution density of the sensor array is $\left(2\times 2\right)/7.5\;\mathrm{cm}\times 6.5\;\mathrm{cm}=0.082\;{\mathrm{cm}}^{-2}$, and the theoretical localization precisions dX=7.5 cm/2=3.75 cm, and dY=6.5 cm/2=3.25 cm. It can be seen that the localization precision of the planar sensor is significantly improved by over two orders of magnitude with the help of the neural network, breaking through the limit of electrode distribution density.

#### 4.2.2. Accuracy Analysis

**Figure 6:**
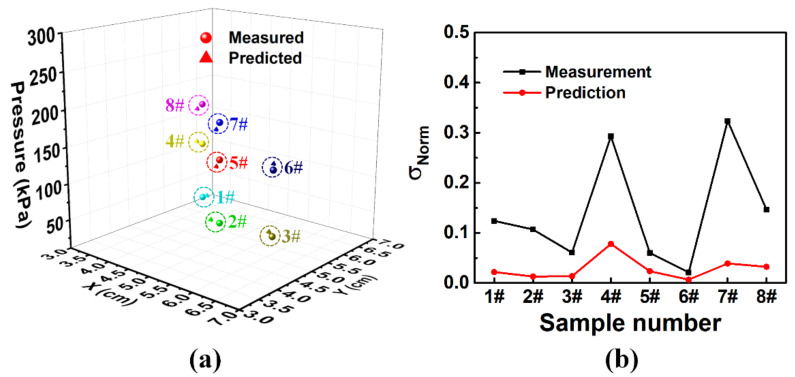
(**a**) Three-dimensional mapping of the predicted and measured results of 8 typical data samples. (**b**) Normalized standard deviations of measurements and predictions of 8 typical test conditions.

The BP-predicted results and the measured results of the eight typical data samples are shown in Figure 6a for accuracy analysis. The deviations between the prediction and measurement of coordinates (*X* and *Y*) were smaller than 0.3 cm, which is also one order of magnitude smaller than the localization precision of the traditional grid-architecture planar pressure sensor array with the same electrode distribution density. The deviation between predicted and measured of pressure was less than 20 kPa.

#### 4.2.3. Repeatability Error Analysis

We also compared the measurement errors to the prediction errors while repetition. Still using the eight test data conditions, we repeated the test of each condition eight times, and the normalized standard deviations of the measured pressure and locations were calculated for each condition, which were defined as the measurement errors. The measured data during the repeatability test of each condition was then input into the neural network model, so that the corresponding predicted results as well as the repetition standard deviation could be obtained. Since the inputs and outputs for the measurement and prediction were in the opposite direction, it was necessary to normalize the measurement error and the prediction error for comparison. The normalized measurement error is expressed by the average normalized standard deviation of the resistance between the four pairs of electrodes during the repeatability test, as is shown in Equation (14); and the normalized prediction error is expressed by the average normalized standard deviation of the predicted coordinates (*X* and *Y*) and the pressure *P*, as shown in Equation (15).

(14) $$\sigma_{norm}\left(meas\right)=\frac{1}{4}\times \left(\begin{array}{c} \frac{1}{\bar{R_{A1}}}\sqrt{\frac{\sum_{i=1}^{m}{\left(R_{A1}-\bar{R_{A1}}\right)}^{2}}{m}}+\frac{1}{\bar{R_{B3}}}\sqrt{\frac{\sum_{i=1}^{m}{\left(R_{B3}-\bar{R_{B3}}\right)}^{2}}{m}}\\ +\frac{1}{\bar{R_{C2}}}\sqrt{\frac{\sum_{i=1}^{m}{\left(R_{C2}-\bar{R_{C2}}\right)}^{2}}{m}}+\frac{1}{\bar{R_{D4}}}\sqrt{\frac{\sum_{i=1}^{m}{\left(R_{D4}-\bar{R_{D4}}\right)}^{2}}{m}} \end{array}\right)$$

(15) $$\sigma_{norm}\left(pred\right)=\frac{1}{3}\times \left(\frac{1}{\bar{X}}\sqrt{\frac{\sum_{i=1}^{m}{\left(X-\bar{X}\right)}^{2}}{m}}+\frac{1}{\bar{Y}}\sqrt{\frac{\sum_{i=1}^{m}{\left(Y-\bar{Y}\right)}^{2}}{m}}+\frac{1}{\bar{P}}\sqrt{\frac{\sum_{i=1}^{m}{\left(P-\bar{P}\right)}^{2}}{m}}\right)$$

where *m* is the total repeating time, which was eight in this work.

The comparison of measurement errors and prediction errors of the eight test conditions is shown in Figure 6b. The results show that the prediction error under each test condition was smaller than the measurement error, indicating that the neural network model has good convergence. Furthermore, the measurement errors under the same pressure at different locations were obviously different, indicating that the in-plane uniformity of our sample was poor. On the other hand, the results also indicated that the planar pressure sensor with poor uniformity could achieve high sensing accuracy for both location and pressure with the help of the BP neural network.

#### 4.2.4. Training Sample Space Analysis

**Figure 7:**
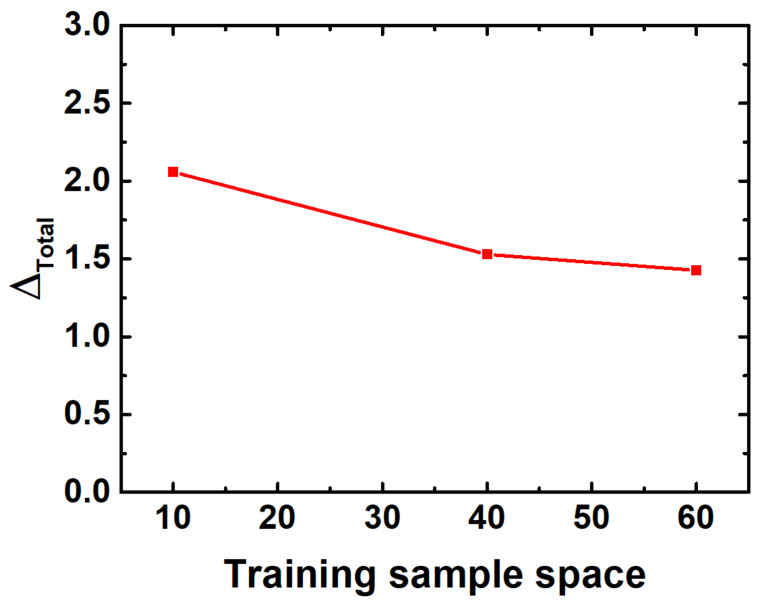
The total relative errors of the predicted results with different number of training samples.

The influence of the number of neural network training samples, or training sample space, on the accuracy of prediction was then investigated. We randomly selected 10, 40, and 60 training data samples from the database to train the neural network and to predict the pressure mappings of the eight typical data samples. The prediction results as well as the comparison to the measurement are shown in Figure 7. The total relative errors of the eight predicted results were calculated as follows, after training with different sample numbers:

(16) $$\Delta_{total}=\sum_{j=1}^{8}\left({\left(\frac{X_{meas}-X_{pred}}{X_{meas}}\right)}^{2}+{\left(\frac{Y_{meas}-Y_{pred}}{Y_{meas}}\right)}^{2}+{\left(\frac{P_{meas}-P_{pred}}{P_{meas}}\right)}^{2}\right)$$

As shown in Figure 7, the total relative error of prediction decreased as the number of training samples increased. Therefore, it is foreseeable that if a large number of training samples are obtained, the prediction accuracy can be further improved till saturation. Limited by time and experiment conditions, only 64 training samples were used in this work. However, as shown above, the prediction reached a surprisingly high accuracy and precision, which is probably sufficient to illustrate the advantages of the design method proposed.

#### 4.2.5. Analysis of the Impact of Material Uniformity

**Figure 8:**
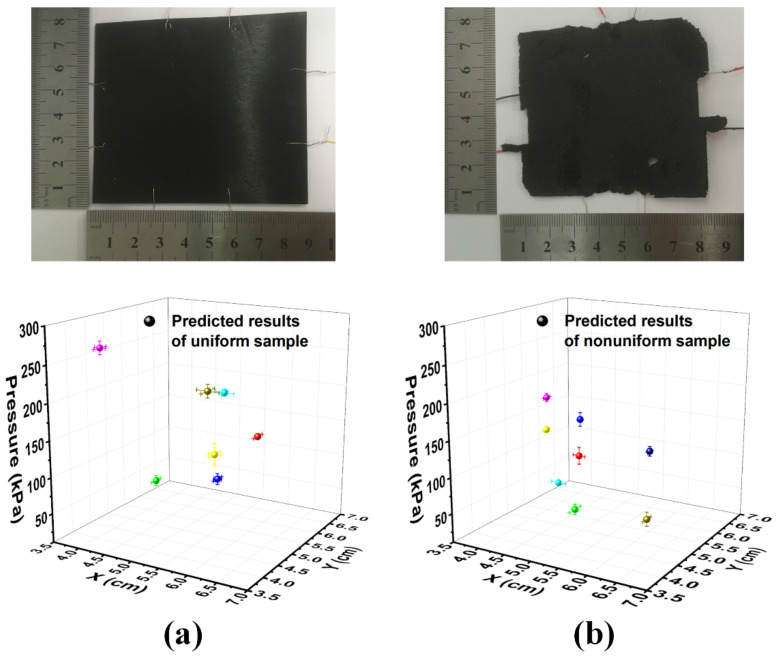
The photos and the predicted results of (**a**) the uniform sample and (**b**) the nonuniform sample. The error bars in the figure indicate the deviation between the predicted results and the measured results.

The impact of the uniformity of the sensing material on the prediction accuracy was also investigated in this work. The uniformity of the sensing material, including but not limited to the composition and thickness, is always a big concern for such planar sensors. However, its impact can be limited to the minimum with the use of the BP neural network. We bought a commercial conductive rubber sheet from Dongguan Ziming Silicone Rubber Products Co., Ltd, Dongguan, China, for comparison with our home-made one (also named as “nonuniform sample”). The carbon black content in the commercial sample was about 40 wt.%, and the uniformity was much better than our home-made sample. The thickness was uniformly 1.5 mm. A similar sensor with four pairs of electrodes were made from the commercial conductive rubber sheet (named as “uniform sample” thereafter), and some typical data samples were predicted with the help of the neural network model using the same testing method as above. Figure 8 shows the comparison between the uniform sample (Figure 8a) and the nonuniform sample (Figure 8b). The symbols in the figure show the prediction results, and the error bars are the deviation from the measurement results. The average prediction deviations of *X*, *Y*, and *P* of the uniform sample were 0.098 cm, 0.119 cm, and 7.45 kPa, respectively, while the average deviations of the nonuniform sample were 0.078 cm, 0.089 cm, and 7.2 kPa, respectively. The results showed no significant difference between the uniform sample and the nonuniform sample, which indicates that the nonuniformity of the sensing material can be well compensated with the help of the neural network. This finding can greatly reduce the uniformity requirements during fabrication and thus the process complexity and the manufacturing cost.

## 5. Application Examples

**Figure 9:**
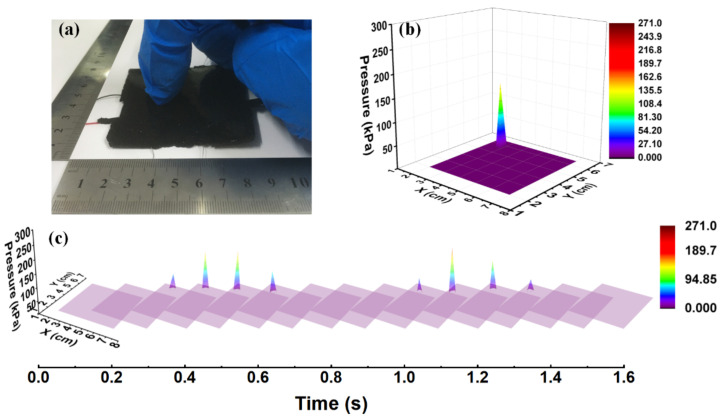
(**a**) The sample was tapped at the upper-left corner, (**b**) the 3D mapping of the tapping pressure and location, and (**c**) the dynamic display of the process of two cycles of tapping within ~1.5 s.

With the help of the BP neural network, the grid-less planar pressure sensors can map the pressure magnitude and location accurately on irregularly-shaped and even non-uniform surfaces, and have extensive application prospects. Combined with time and other parameters, the grid-less planar pressure sensors can realize more pressing pattern recognition and be more intelligent. Here we show a typical application of pattern recognition to differentiate the “tap” and “press” mode at any random location. As shown in Figure 9, the sample was tapped at the upper-left corner of the sample (Figure 9a), the 3D mapping of the tapping pressure and location (3.5 cm, 5.5 cm) is shown in Figure 9b, and the dynamic display of the process of two cycles of tapping within ~1.5 s is shown in Figure 9c. The predicted results clearly show accurate localization and magnitude of the tapping in real time.

**Figure 10:**
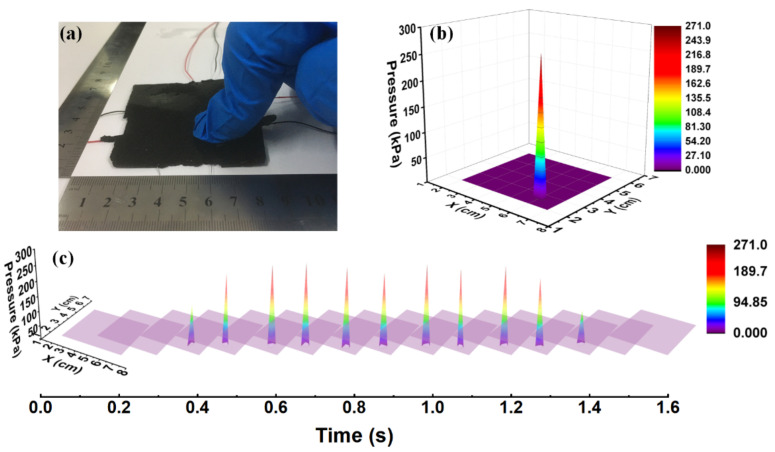
(**a**) The sample was pressed at the lower-right corner, (**b**) the 3D mapping of the pressing pressure and location, and (**c**) the dynamic display of the process of one cycle of pressing within ~1.5 s.

As shown in Figure 10, the sample was pressed for a period at the lower-right corner (Figure 10a), the 3D mapping of the pressure and location (6 cm, 2.5 cm) is shown in Figure 10b, and the dynamic display of the process of one cycle of pressing within ~1.5 s is shown in Figure 10c. The predicted results clearly show accurate localization and magnitude of the pressing in real time.

## 6. Conclusions

A general design method of the grid-less planar sensor was proposed and verified in this work, with the help of the BP neural network. Compared to the traditional grid-architecture planar sensor array for pressure and location with the same electrode distribution density, the prediction precision can be over two orders of magnitude higher, the accuracy can be one order of magnitude higher, and the repetition accuracy of prediction was better than that of measurement. The prediction accuracy of this method can be further improved by using more advanced neural network algorithms and/or by using a larger number of training data. In addition, this method works well for irregularly-shaped sensors, and the prediction accuracy was proved to be irrelevant to the uniformity of the sensing material. Based on the above research results, the grid-less planar sensor can realize high-precision pressure mapping with a simpler structure and material design, and can have extensive application prospects in many fields, including bionic electronic skin.

## Figures and Tables

**Table 1:** The number, coordinates, and pressure values of 8 typical test data samples.

Selected Point (#)	Coordinates		Pressure (kPa)
	X (cm)	*Y* (cm)	
**1**	6.5	4.5	58.6
**2**	5.4	4.4	59.2
**3**	4.3	5.2	61.4
**4**	6.5	4.5	145.4
**5**	5.4	4.4	144.3
**6**	4.3	5.2	137.9
**7**	5.4	4.4	193.3
**8**	4.3	5.2	193.5
